# Absence of plastin 1 causes abnormal maintenance of hair cell stereocilia and a moderate form of hearing loss in mice

**DOI:** 10.1093/hmg/ddu417

**Published:** 2014-08-14

**Authors:** Ruth Taylor, Anwen Bullen, Stuart L. Johnson, Eva-Maria Grimm-Günter, Francisco Rivero, Walter Marcotti, Andrew Forge, Nicolas Daudet

**Affiliations:** 1 Centre for Auditory Research, UCL Ear Institute, University College London, London, UK; 2 Department of Biomedical Science, University of Sheffield, Sheffield, UK and; 3 Centre for Cardiovascular and Metabolic Research, The Hull York Medical School, University of Hull, Hull, UK

## Abstract

Hearing relies on the mechanosensory inner and outer hair cells (OHCs) of the organ of Corti, which convert mechanical deflections of their actin-rich stereociliary bundles into electrochemical signals. Several actin-associated proteins are essential for stereocilia formation and maintenance, and their absence leads to deafness. One of the most abundant actin-bundling proteins of stereocilia is plastin 1, but its function has never been directly assessed. Here, we found that plastin 1 knock-out (*Pls1* KO) mice have a moderate and progressive form of hearing loss across all frequencies. Auditory hair cells developed normally in *Pls1 KO*, but in young adult animals, the stereocilia of inner hair cells were reduced in width and length. The stereocilia of OHCs were comparatively less affected; however, they also showed signs of degeneration in ageing mice. The hair bundle stiffness and the acquisition of the electrophysiological properties of hair cells were unaffected by the absence of plastin 1, except for a significant change in the adaptation properties, but not the size of the mechanoelectrical transducer currents. These results show that in contrast to other actin-bundling proteins such as espin, harmonin or Eps8, plastin 1 is dispensable for the initial formation of stereocilia. However, the progressive hearing loss and morphological defects of hair cells in adult *Pls1 KO* mice point at a specific role for plastin 1 in the preservation of adult stereocilia and optimal hearing. Hence, mutations in the human *PLS1* gene may be associated with relatively mild and progressive forms of hearing loss.

## INTRODUCTION

The mechanosensory ‘hair’ cells of the inner ear derive their name from the organized bundle of erect, cytoskeletal projections from their apical surface. The bundle is comprised of stereocilia arranged in rows of increasing height, anchored within an actin-rich structure called the cuticular plate. Movements of inner ear fluids induced by sound vibrations (in the cochlea) or changes in head position (in the vestibular system) deflect stereocilia and stimulate hair cell responses, a process known as ‘mechanotransduction’ (1).

**Figure 1.**
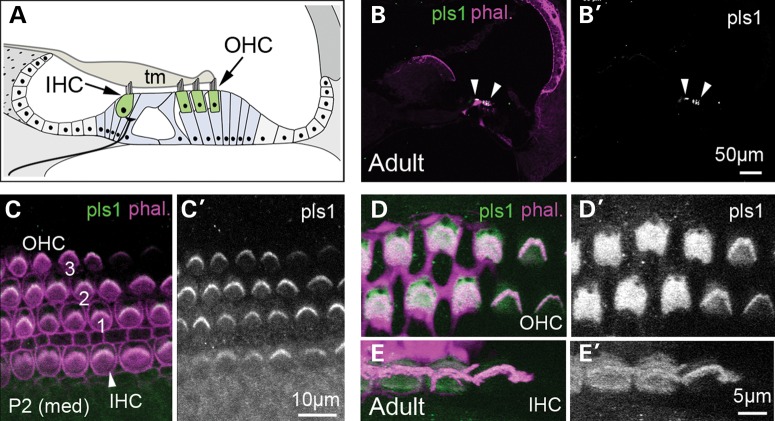
The organ of Corti and expression of plastin 1 in the mouse inner ear. (**A**) Schematic transverse view of the organ of Corti. The two types of auditory hair cells, the IHCs and OHCS, rest on supporting cells, and their stereociliary bundles are in contact with the tectorial membrane (tm). The IHCs are contacted by the majority of afferent nerve fibres and are the primary receptors of auditory signals conveying information to the brain. The OHCs have unique electromotile properties and play an important role in hearing sensitivity and frequency discrimination by locally amplifying sound-elicited vibrations of the organ of Corti. (**B**–B′) Low-magnification transverse view of the adult mouse organ of Corti. Plastin 1 immunoreactivity is only detected at the apical surfaces of hair cells (arrowheads). (**C**–E′) Surface preparation of the organ of Corti of P2 (C–C′) and adult (**D**–E′) mice immunostained for plastin 1 and counterstained with fluorescently labelled phalloidin. Plastin 1 is present in immature stereocilia, and its expression is maintained in the stereocilia and cuticular plate of both types of auditory hair cells at adult stages.

Like microvilli, the stereocilia contain a central core of densely packed parallel actin filaments of the same polarity, the number and length of which vary proportionally to stereocilia size (2–5). This actin core is thought to confer rigidity to stereocilia, which upon mechanical stimulation behave like stiff rods that pivot around their tapered base (6,7). Three classes of actin-bundling proteins, belonging to the espin, fascin and plastin families, are present in stereocilia. Distinct espin isoforms are expressed in developing and mature stereocilia (8), and mutations in the *ESPN/Espn* gene cause a profound form of congenital deafness in humans (9,10) and in *jerker* mice (11). In the absence of espin, stereocilia fail to widen and degenerate soon after their initial formation, suggesting that espin is required for the lateral apposition of parallel actin bundles within stereocilia and their consequent increase in diameter (12,13). Fascin2 appears during the final stages of stereocilia elongation and is concentrated at the tips of the taller stereocilia (14). Mutations of the *Fscn2* gene cause a progressive degeneration of stereocilia and an early onset age-related hearing loss (ahl8) in DBA/2J mice, which also carry a mutation in *cadh23* (14). Of the plastins, plastin 3 is transiently expressed during the formation of stereocilia in immature hair cells (15), whereas plastin 1, the homologue of chicken fimbrin (16), is present in the stereocilia and cuticular plate of developing and mature hair cells (Fig. 1 and Supplementary Material, Fig. S1) (17–21). Biochemical studies have shown that plastin 1 is one of the most abundant proteins of stereocilia (14,22,23), yet its actual contribution to stereocilia formation and function has not been directly assessed.

Here, we report that *plastin 1* knock-out (*Pls1* KO) mice (24) have a moderate and progressive form of hearing loss associated with defects in stereocilia morphology. These defects are surprisingly subtle compared with the major structural malformations resulting from the loss of the other actin-bundling proteins of stereocilia, suggesting that plastin 1 is specifically involved in the preservation of the parallel actin bundles in mature stereocilia. Hence, mutations in the human *PLS1* gene could be associated with mild and progressive forms of hearing loss.

## RESULTS

### Plastin 1-deficient mice have a moderate form of hearing loss across all frequencies that is not caused by hair cell death

**Figure 2.**
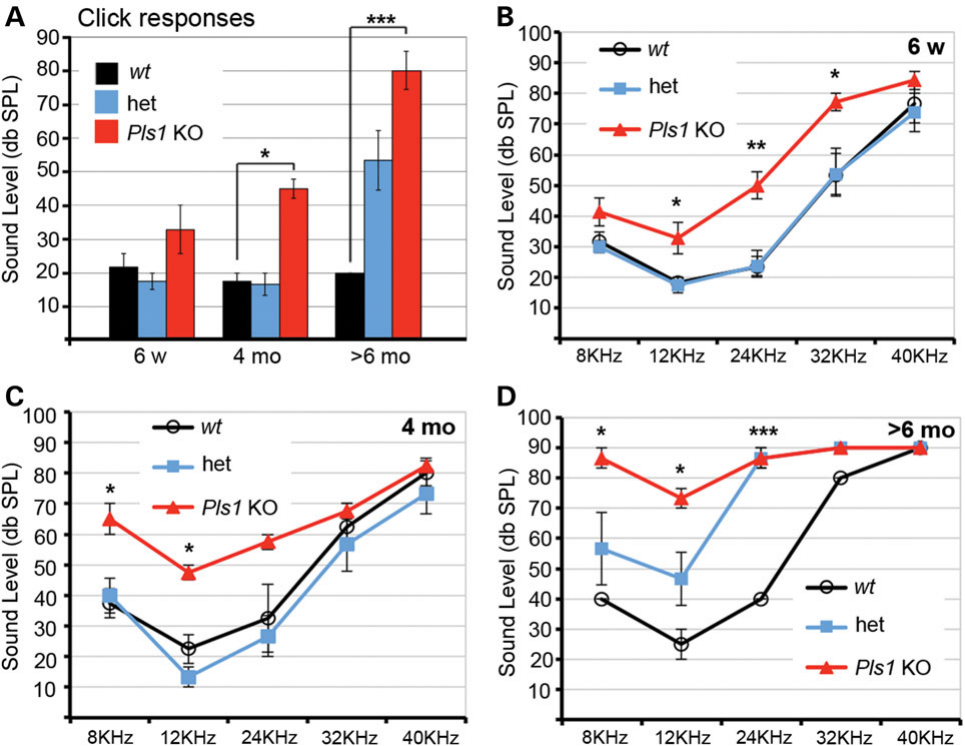
*Pls1 KO* mice have a moderate and progressive form of hearing loss. Auditory brainstem responses to click (**A**) stimuli and to tone pips (**B**–**D**) at 8, 12, 24, 32 and 40 kHz at different ages in control (wt), het and *Pls1* KO mice. *P*-values for Tukey–Kramer multiple comparisons test, *P* < 0.05 (*), *P* < 0.01 (**) and *P* < 0.001 (***). Significant differences between *Pls1* KO and wt thresholds are indicated on (B–D). Error bars represent SEM.

To determine the potential importance of plastin 1 for hearing, we first recorded auditory brainstem responses (ABR) to click and tone pip stimuli in anaesthetized wild-type (wt), heterozygous (het) and *Pls1 KO* mice (Fig. 2). In young adult mice (6 weeks), analysis of ABR data in response to click stimuli (Fig. 2A) showed no significant difference in hearing thresholds between wt, het and *Pls1* KO mice. In 4-month-old animals, thresholds were significantly raised in *Pls1* KO compared with wt and het (*P* < 0.05). In animals older than 6 months, hearing thresholds were significantly raised in Pls1 KO compared with wt (*P* < 0.001) but there were no significant differences between *Pls1* KO and het, or between het and wt. Comparison of the results for animals of the same genotype showed a very significant difference (*P* < 0.001) for responses to click stimuli between the young *Pls1* KO and *Pls1* KO mice older than 6 months. Equally, whereas there was no significant threshold shift between the young and oldest wt animals, there was a significant (*P* < 0.01) threshold shift between the youngest and oldest het animals.

Analysis of responses to tone pip stimuli showed significant difference at 12, 24 and 32 kHz between 6-week-old wt (but not het) and *Pls1* KO (Fig. 2B) mice. In 4-month-old mice, *Pls1* KO showed a significant difference to wt at 8 and 12 kHz (Fig. 2C), but not at 24 kHz, although their hearing thresholds remain elevated. In mice aged 7–9 months, hearing thresholds were elevated for het animals for all frequencies tested between 8 and 32 kHz, and even greater thresholds shifts were apparent for *Pls1* KO when compared with wt (Fig. 2D).

Altogether, these results show that the absence of plastin 1 causes a moderate form of hearing loss in young adult mice (10–20 dB), progressing to a severe loss (40–50 dB) with age.

**Figure 3.**
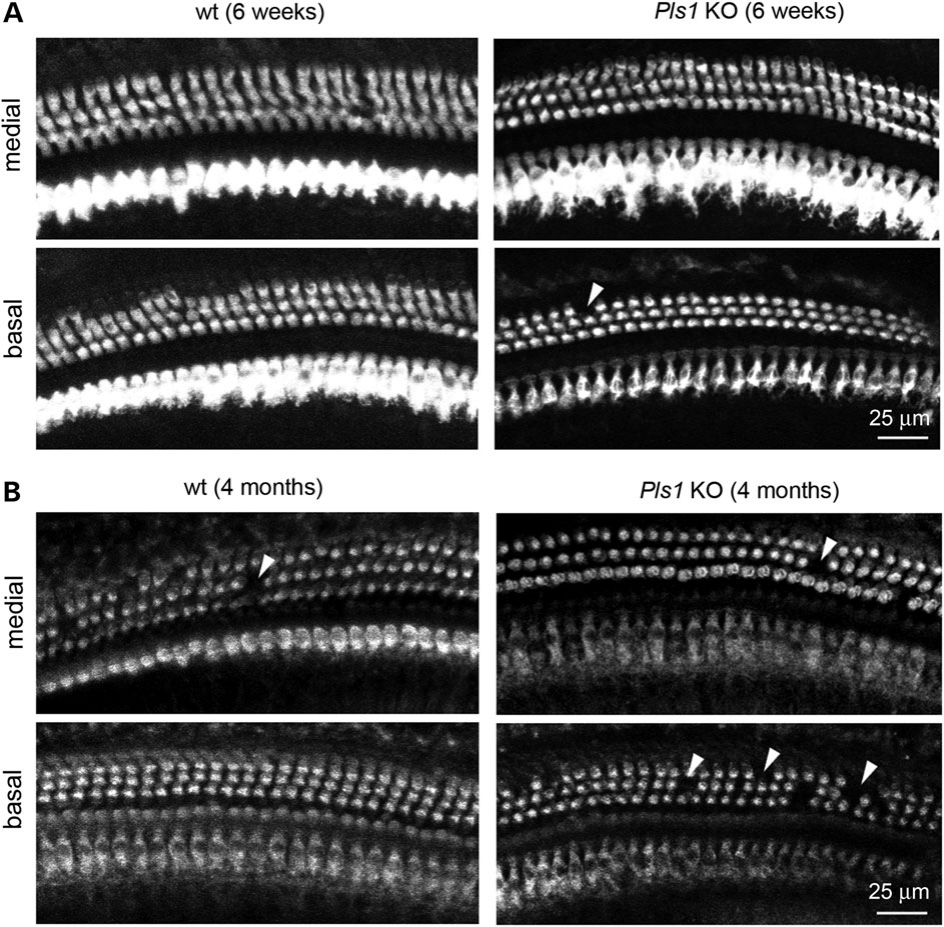
Hair cell loss is not responsible for the hearing deficit in *Pls1 KO* mice. (**A**) Whole-mount, surface views of the organ of Corti of 6-week-old wt and *Pls1* KO mice immunostained for parvalbumin. The vast majority of hair cell bodies are present in the medial and basal turn of the cochlea. Arrowhead points to the site of one missing OHC in the *Pls1* KO sample. (**B**) Surface views of the organ of Corti of 4-month-old wt and *Pls1* KO mice immunostained for myosin-VIIa . The IHC are well preserved in both the *Pls1* KO and wt mice, but some OHCs are missing in wt and *Pls1* KO mice (arrowheads), with increased frequency of OHC losses in the basal turns of the cochlea.

To determine whether the auditory deficit in *Pls1* KO mice might be due to hair cell loss, we analysed whole-mount or sectioned preparations of the organ of Corti labelled with phalloidin and hair cell-specific antibodies. In 6-week-old animals (Fig. 3A), the vast majority of inner (IHCs) and outer (OHCs) hair cells were present at all levels of the cochlea regardless of genotype, indicating that the absence of plastin 1 did not impair hair cell formation or survival. In 4-month-old animals (Fig. 3B), most IHCs and OHCs were present although a higher degree of OHC loss was noticed, particularly in the basal turn of *Pls1* KO mice. These data indicate that the hearing loss found at all frequencies in young adult *Pls1 KO* animals is not consecutive to hair cell death.

### Absence of plastin 1 causes morphological defects in mature stereocilia

**Figure 4.**
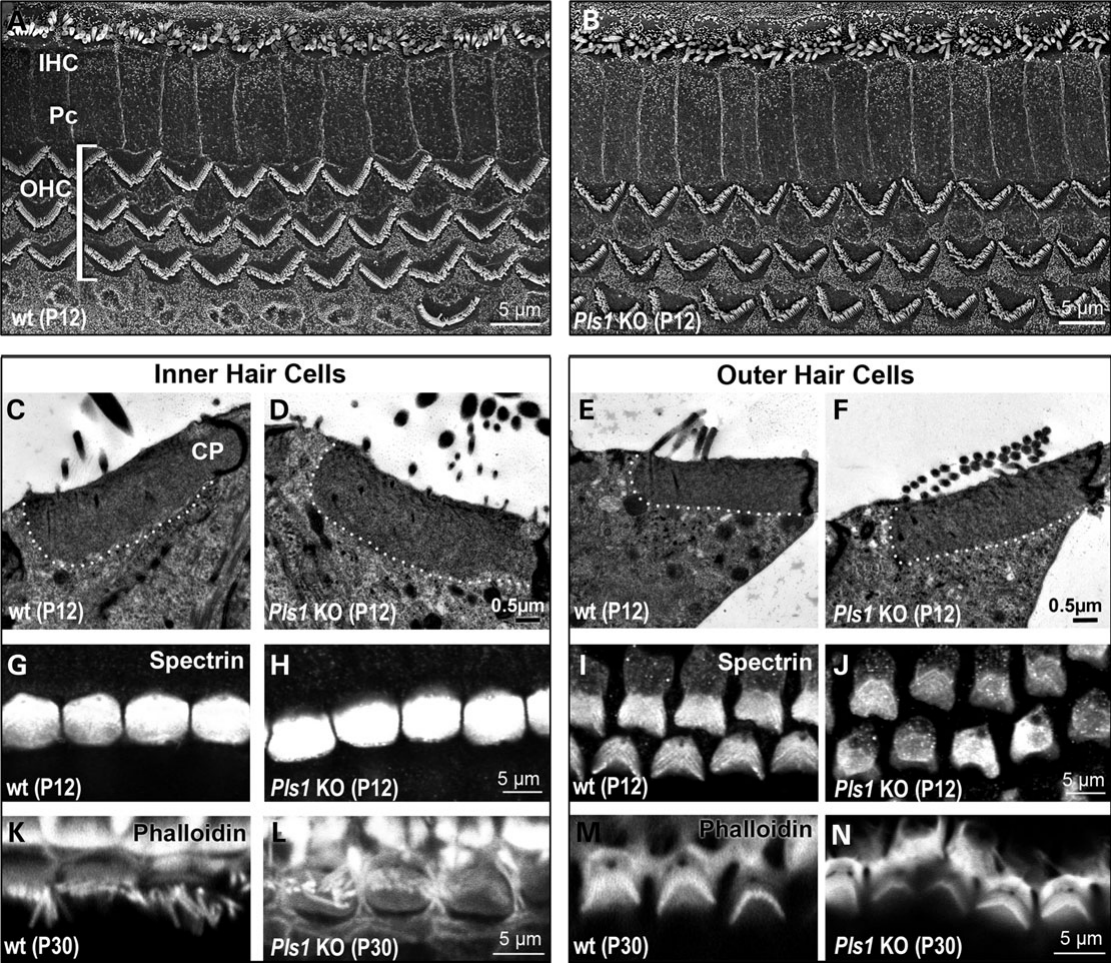
Auditory hair cells develop normally in young *Pls1 KO* mice. (**A** and **B**) Scanning electron micrographs of the surface of the organ of Corti in the medial turn of the cochlea of P12 wt (A) and *Pls1 KO* (B) mice. (**C**–**F**) Transmission electron micrographs of the apical region of the IHC and OHC in P12 wt and Pls1 KO mice. The contour of the cuticular plate (CP) is outlined by a white dotted line. (**G**–**J**) Surface views of the IHCs and OHCs of P12 wt and Pls1 KO mice immunostained for spectrin, a marker of the CP. (**K**–**N**) Surface views of the IHCs and OHCs of P30 wt and Pls1 KO mice stained with fluorescent phalloidin. The F-actin content of stereocilia and the CP appears similar in wt and *Pls1 KO* hair cells.

To examine in greater detail stereocilia and hair cell morphology in *Pls1* KO mice, we used scanning (SEM) or transmission (TEM) electron microscopy. In young (P12) animals, the overall organization of the organ of Corti was unchanged in *Pls1* KO mice compared with wt littermates (Fig. 3A and B). The stereociliary bundles of hair cells displayed a characteristic straight (for IHC) or V-shape (for OHC) and were of uniform orientation. The cuticular plates of plastin 1-deficient IHCs and OHCs developed normally and showed similar thickness, spectrin expression and actin content to those of wt hair cells (Fig. 4C–N).

**Figure 5.**
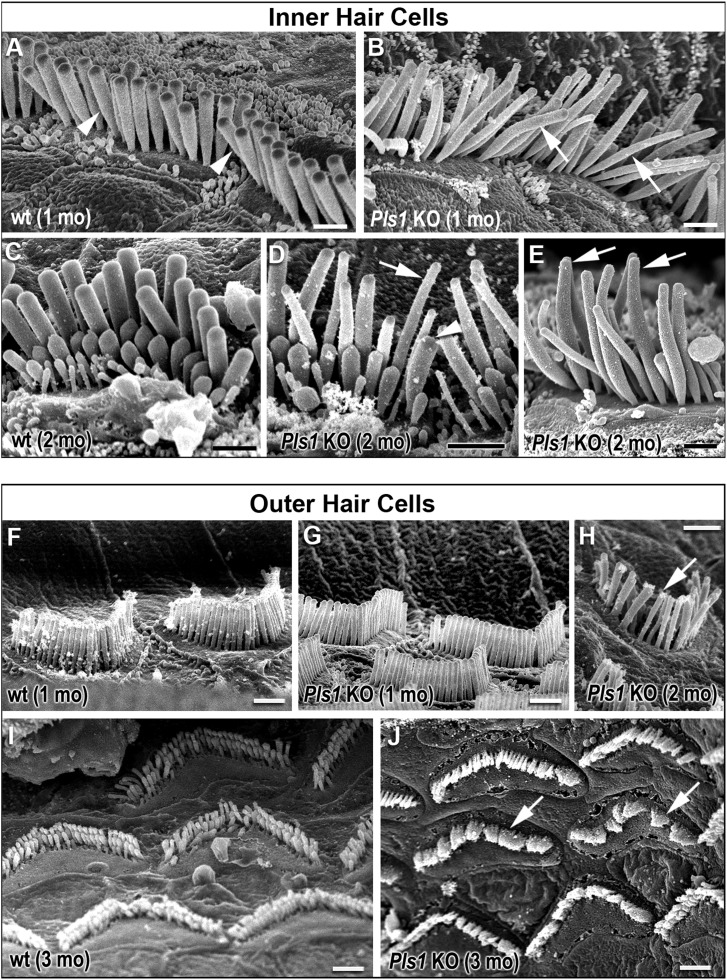
Morphological defects of stereocilia in adult *Pls1* KO mice. (**A**–**E**) Scanning electron micrographs of IHCs in wt and *Pls1* KO mice. Compared with those of P30 wt littermates (arrowheads in A), the stereocilia of *Pls1* KO mice show a reduced width and abnormal bending (arrows in B). Similar defects are visible at 8 weeks (C–E). Note that the same IHC can have a mixture of thin (arrow) and normal (arrowhead) stereocilia (D). A distal tapering is also visible in some of the stereocilia of *Pls1* KO (arrows in E). (**F**–**J**) Scanning electron micrographs of OHCs in wt and *Pls1* KO mice. (F and G) At P30, the stereocilia of OHCs were very much less affected than those of IHCs in Pls1 KO mice and looked normal. There was no evidence of reduced width or abnormal bendiness. (H) At 8 weeks, some defects such as fusion of neighbouring stereocilia (arrow) were occasionally visible. (I and J) At 12 weeks, the stereocilia of *Pls1* KO OHCs exhibited increased fusion and defects in organization (arrows) compared with those of wt OHCs; both images were taken from the basal turn of the cochlea. Scale bars = 1 µm.

However, at adult stages, the stereocilia of IHCs of *Pls1* KO mice showed striking morphological defects (Fig. 5 and Table 1). They were frequently thinner, unusually bent (Fig. 5B) and of variable length within the same row (Fig. 5D and 5E). Within the same bundle, a mixture of normal-looking and abnormal stereocilia was frequently observed. The longest row of stereocilia showed an average reduction in width of 10–20% and the minimum width of individual stereocilia in *Pls1* KO IHCs (0.15 µm) was much reduced when compared with that in wt animals (0.32 µm). Some stereocilia were reduced in width along their entire length, whereas in others, the distal end of the stereocilium was noticeably thinner than the proximal end (see for example Fig. 5E). Although the mean height of the row of longest stereocilia was not significantly reduced at any age examined up to 4 months (Table 1), there was a noticeable variability in the length of the longest stereocilia within the bundles of individual IHCs, which became more apparent with age (compare Fig. 5B with Fig. 5D and E; see also Supplementary Material, Fig. S2). These data indicate that the absence of plastin 1 caused structural defects in IHC stereocilia that affected their width and length, and perhaps their rigidity.

**Table 1.** Measurements of the width and height of the longest stereocilia of IHCs at different turns of the cochlea in 13 adult het and 15 Pls1 KO animals

	het			*Pls1* KO		
	Mean (µm) [SD]	Range (µm)	*n*	Mean (µm) [SD]	Range (µm)	*n*
Width						
Base	0.41 [0.04]	0.32–0.48	93	0.36 [0.05]*	0.15–0.46	150
Mid-base	0.43 [0.03]	0.36–0.49	81	0.31 [0.06]*	0.15–0.49	145
Mid-apex	0.41 [0.03]	0.35–0.47	68	0.33 [0.07]*	0.17–0.44	78
Height						
Base	2.2 [0.15]	1.54–2.45	41	2.0 [0.3]	1.5–2.8	117
Mid-apex	3.3 [0.5]	2.69–3.85	81	3.6 [0.5]	2.13–4.85	55

SD, standard deviation; T-test *P*-values < 0.01 (*) are indicated. *n* = number of stereocilia analysed.

In contrast, the stereociliary bundles of OHCs were similar in overall appearance and organization to those of wt and het littermates (Fig. 5F and G). They formed two to three rows of graded height and were of similar size within the same row, regardless of the age and genotype examined (Table 1). However, by 2 months, there was some evidence of stereocilia fusion and variations in width on some OHCs at least (Fig. 5H–J), suggesting that plastin 1 is necessary for the maintenance of stereocilia in both types of auditory hair cells.

### The organization of actin filaments is not drastically modified by the absence of plastin 1

**Figure 6.**
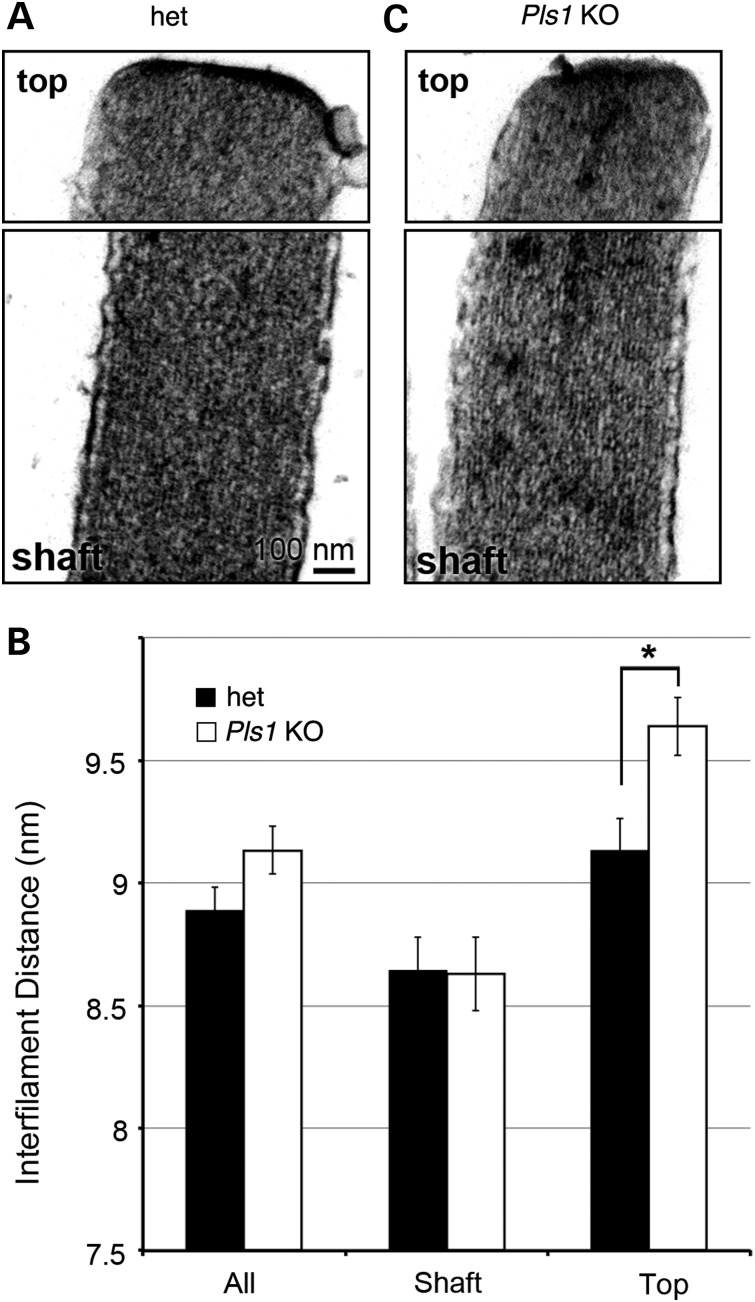
Organization of the actin filaments in the stereocilia of *Pls1 KO* mice. (**A** and **B**) Transmission electron micrographs of the top and shaft region of the IHC stereocilia in adult (4 month and older) het and *Pls1 KO* mice. (**C**) Mean values of the interfilament distance within the stereocilia of het and *Pls1 KO* mice. Mean values are shown for all measurements, and those obtained from either the shaft or the top region (within 300 nm of the tip) only; 250 measurements were taken from 5 different stereocilia for each genotype; error bars represent the standard error of the mean. The interfilament distance was significantly increased in the top region of stereocilia of *Pls1 KO* mice compared with het mice (*P*-value = 0.004; unpaired *T*-test with Welch's Correction).

To determine whether the absence of plastin 1 might affect the structural organization of the parallel actin bundles within stereocilia, high-resolution images of thin sections of IHCs stereocilia from adult het and *Pls1 KO* mice were collected (Fig. 6A and B). The mean distance between actin filaments was measured (Fig. 6C) at two different levels of stereocilia: the top region (within 300 nm of the tip) and the shaft region located more basally. At the level of the shaft, there was no significant difference in the packing density of actin filaments in abnormal-looking stereocilia of *Pls1* KO (mean = 8.628 nm, SD = 2.36; *n* = 250 measurements) compared with het (mean = 8.644 nm, SD = 2.154; *n* = 250) samples. This suggests that the reduction in width of IHC stereocilia is primarily due to a reduction in the number of actin filaments they contain and not to drastic changes in their packing. In the top region, however, the interfilament distance was slightly but significantly (*P* < 0.05) increased in *Pls1* KO (mean = 9.638 nm, SD = 1.851; *n* = 250, five stereocilia) compared with het (mean = 9.134 nm, SD = 2.049; *n* = 250, five stereocilia) samples, suggesting that plastin 1 may have a particular importance for F-actin crosslinking at this site, where the plus end of actin filaments is located.

### Basolateral membrane properties of inner hair cells develop normally in *Pls1* KO mice

**Figure 7.**
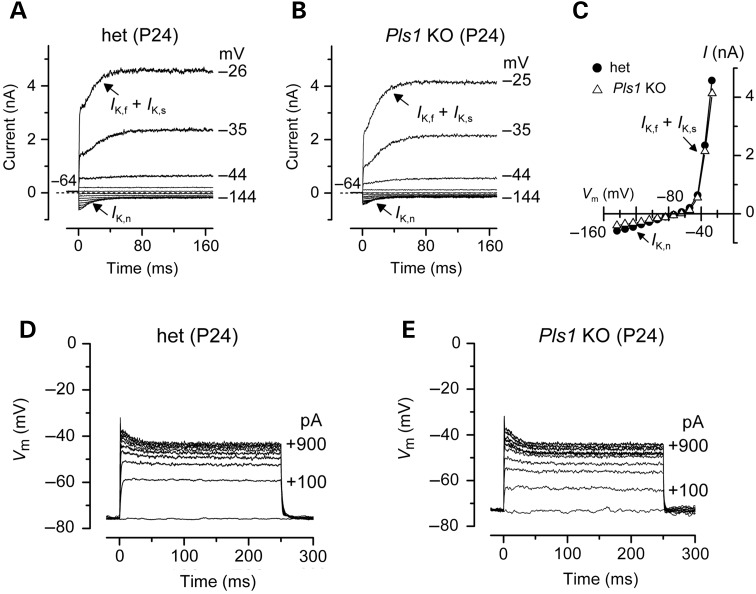
Current and voltage responses from IHCs of plastin 1 mice. (**A** and **B**) K^+^ currents recorded from mature het and *Pls1* KO P24 IHCs were elicited by depolarizing voltage steps (10-mV nominal increments) from –144 mV to more depolarized values from the holding potential of –64 mV. The K^+^ currents characteristic of adult IHCs, *I*_K,f_ and *I*_K,n_ were similarly expressed in both genotypes. (**C**) Steady-state current–voltage curves for the total K^+^ current in het (*n* = 5) and *Pls1* KO (*n* = 5) P24 IHCs. (**D** and **E**) Voltage responses to different current injections recorded from a het and a *Pls1* KO IHC.

We next asked whether mature (P24) IHCs from *Pls1* KO mice had normal biophysical properties, such as a rapidly activating large conductance Ca^2+^-activated K^+^ current (*I*_K,f_) and a current carried by KCNQ4 channels (*I*_K,n_) with an unusually hyperpolarized activation range (25). Depolarizing and hyperpolarizing voltage steps from the holding potential of −64 mV elicited similar voltage-dependent outward K^+^ currents in all IHCs tested from het and *Pls1* KO mice (Fig. 7A–C). *I*_K,f_ and *I*_K,n_ were expressed with a similar amplitude in IHCs from both genotypes (Table 2). Voltage responses as well as all other biophysical properties (Table 2) were also similar between het and *Pls1* KO IHCs (Fig. 7D and E), indicating that the absence of plastin 1 has no impact on the overall acquisition of the biophysical characteristics of IHCs.

**Table 2.** Electrophysiological properties of IHCs in adult het and Pls1 KO mice

	het	*Pls1* KO
Membrane capacitance (pF)	13.0 ± 0.6 (5)	12.6 ± 1.0 (5)
Resting potential (mV)	−74.2 ± 0.7 (5)	−71.5 ± 1.7 (4)
*I*_K_ at 0 mV (nA)	20.4 ± 2.3 (5)	20.0 ± 0.8 (5)
*I*_K,f_ at −25 mV (nA)	3.5 ± 0.4 (5)	3.0 ± 0.4 (5)
*I*_K,n_ at −124 mV (pA)	291 ± 37 (5)	231 ± 32 (4)

Values are means ± SEM; number of hair cells is in parentheses. *I*_K_ = delayed rectifier K^+^ current; *I*_K,n_ = negatively activating K^+^ current carried by KCNQ4 channels; *I*_K,f_ = Ca^2+^-activated K^+^ current. Numbers of IHCs analysed are indicated in brackets (*n*).

### Mechanoelectrical transducer currents exhibit minor changes in their adaptation properties in *Pls1* KO mice

**Figure 8.**
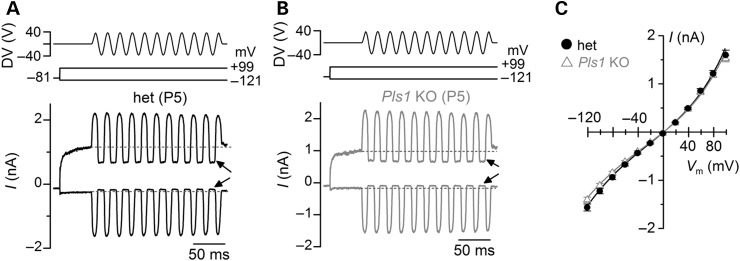
Mechanotransducer currents in OHCs from *Pls1* KO mice. (A, B) Saturating transducer currents recorded from a P5 het (**A**) and *Pls1* KO (**B**) apical-coil OHC by applying a 50-Hz sinusoidal force stimuli to the hair bundles at the potential of −121 mV and +99 mV. The driver voltage (DV) signal of ±40 V to the fluid jet is shown above the traces (negative deflections of the DV are inhibitory). The arrows indicate the closure of the transducer channels, i.e. disappearance of the resting current, during inhibitory bundle displacements at −121 mV and +99 mV, respectively. Dashed lines indicate the holding current. (**C**) Peak-to-peak current–voltage curves were obtained from 7 het and 12 *Pls1* KO OHCs (P5–P8) using 1.3 mm extracellular Ca^2+^. The fits through the data are according to Equation 1 (see Material and methods) with values: het *k* = 418 ± 33, *V*_r_ = 1.3 ± 0.3 mV, *V*_s_ = 38 ± 2 mV and *γ* = 0.42 ± 0.01; *Pls1* KO *k* = 418 ± 40, *V*_r_ = 1.1 ± 0.4 mV, *V*_s_ = 40 ± 3 mV and *γ* = 0.41 ± 0.01.

Besides its role in maintaining the morphology and structure of adult hair cell stereocilia, plastin 1 may be important for mechanoelectrical transduction. To test this, mechanoelectrical transducer (MET) currents were recorded from P5–P8 apical-coil OHCs by displacing their hair bundles in the excitatory and inhibitory direction using a piezo-driven fluid-jet (26,27). The apparent overall steady-state stiffness of the hair bundle measured in het OHCs (6.3 ± 0.6 mN/m, *n* = 16) was similar to that of *Pls1* KO cells (8.3 ± 1.0 mN/m, *n* = 11) and comparable with that measured from wild-type mouse OHCs in organotypic cultures (28). Upon moving the bundles towards the taller stereocilia (i.e. in the excitatory direction) and at negative membrane potentials, a large inward MET current could be elicited in OHCs from both het and *Pls1* KO mice (Fig. 8A and B). The maximum MET current was found to be similar between het (−937 ± 46 pA at −81 mV, *n* = 7) and KO cells (−843 ± 34 pA at −81 mV, *n* = 12). Any resting current flowing through open MET channels in the absence of mechanical stimulation was reduced when bundles were moved towards the shorter stereocilia (i.e. in the inhibitory direction) in all het and *Pls1* KO OHCs (Fig. 8A and B, arrows). Because the MET current reverses near 0 mV, it became outward when excitatory bundle stimulation was applied during voltage steps positive to its reversal potential (Fig. 8A–C). At positive potentials, the larger resting transducer current (e.g. at +99 mV in Fig. 8A and B: arrows) is due to an increased open probability of the transducer channel resulting from a reduced driving force for Ca^2+^ influx (29,30). The above-mentioned results indicate that the biophysical properties of the transducer channel, including the presence of a resting current, are not affected by the absence of plastin 1.

**Figure 9.**
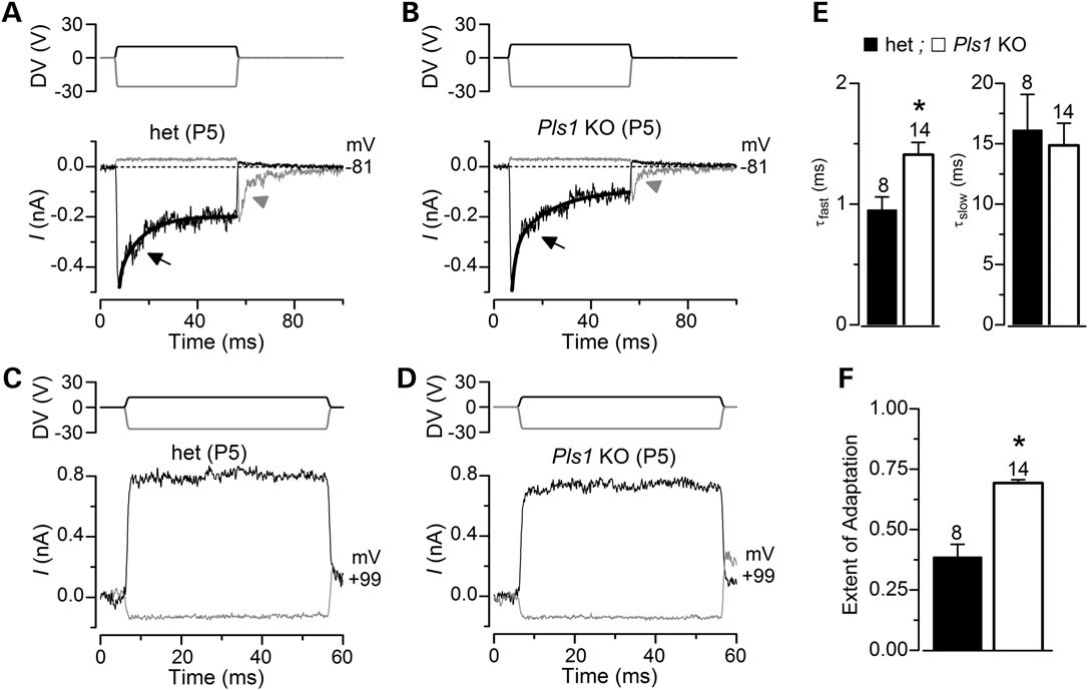
Adaptation properties of the MET current in *Pls1* KO OHCs. (**A** and **B**) Driver voltages to the fluid jet (top) and transducer currents recorded at –81 mV (bottom) from a het and a *Pls1* KO OHC, respectively. At –81 mV, positive DVs (excitatory direction) elicited inward transducer currents that declined or adapted over time in OHCs (arrows). Current decline was best fitted with two time constants (thick line superimposed on the currents): control *τ*_fast_ 1.2 ms, *τ*_slow_ 18.6 ms; knock-out *τ*_fast_ 1.4 ms, *τ*_slow_ 12.8 ms. A small transducer current was present at rest (before t = 0) and inhibitory bundle displacements turned this off. Upon termination of the inhibitory stimulus, the transducer current in het and *Pls1* KO OHCs showed evidence of rebound adaptation (arrowheads). (**C** and **D**) Driver voltages to the fluid jet (top) and transducer currents recorded at +99 mV (bottom) from a het and a *Pls1* KO OHC, respectively. Note that all manifestations of transducer current adaptation (current decline during excitatory stimuli and rebound following inhibitory stimuli) were absent at +99 mV and the resting current increased. (**E**) Average fast and slow time constants (*τ*) used to fit the onset adaptation to excitatory displacement at –81 mV in het and *Pls1* KO OHCs (P5–P8), including the cells shown in (A) and (B). (**F**) Extent of adaptation to excitatory displacement from the same cells used in (E).

We then investigated whether the absence of plastin 1 affected the adaptation properties of the MET current by stimulating the hair bundles of OHCs by mechanical step stimuli instead of sinusoids. In both het and *Pls1* KO OHCs, excitatory bundle movements with non-saturating stimuli elicited rapid inward currents at a holding potential of –81 mV that declined or adapted over time (Fig. 9A and B, arrows). Inhibitory hair bundle stimulation shut off the small fraction of the current flowing at rest, and the offset of large inhibitory steps caused a transient rebound (downward current dip: Fig. 9A and B, arrowheads). All these manifestations of MET current adaptation were absent when stepping the membrane potential to +99 mV (Fig. 9C and D), which prevents or strongly reduces Ca^2+^ entry into the MET channels. This is consistent with Ca^2+^ entry driving adaptation as previously demonstrated in hair cells from lower vertebrates (29,31), but somehow different from recent findings in rat OHCs (32). At −81 mV, the onset adaptation for excitatory stimuli was best fitted with two time constants (*τ*_fast_ and *τ*_slow_) in both het and *Pls1* KO OHCs (Fig. 9A and B, arrows) with *τ*_fast_ (Fig. 9E, left panel), but not *τ*_slow_ (Fig. 9E, right panel), being significantly faster in het than in *Pls1* KO OHCs (*P* < 0.05). The extent of adaptation for non-saturating excitatory stimuli (Fig. 9A and B) was also significantly increased (*P* < 0.0001) in *Pls1* KO compared with het OHCs (Fig. 9F). Although these data were collected on pre-hearing OHCs only, they indicate that plastin 1 is dispensable for mechanotransduction. However, the subtle but significant slowing of the fast adaptation, but overall increase in its extent, to mechanical stimuli suggests that even in the absence of any visible morphological defect, some of the functional properties of stereocilia differ in plastin 1 deficient hair cells.

## DISCUSSION

Plastin 1 is one of the most abundant proteins of developing and mature stereocilia and has long been thought to be an essential crosslinker of actin filaments in this organelle. Our results show that surprisingly, its absence does not cause dramatic structural abnormalities of stereocilia such as those seen in *jerker* mice lacking espin, the other major actin-bundling protein in stereocilia (33). We also found that the biophysical and electrophysiological properties of young *Pls1* KO hair cells are almost unchanged. Nevertheless, the absence of plastin 1 does cause a hearing impairment in adult mice. The *Pls1* KO mice are on a C57BL/6 genetic background, which is known for its increased susceptibility to age-related hearing loss owing to the *Cdh23^ahl^* allele and additional genetic factors (34,35). In these mice, hair cell loss starts at ∼1 month in the basal regions of the cochlea (36), and we cannot exclude the possibility that the hearing loss in *Pls1* KO mice is exacerbated by genetic interactions with *Cdh23*, in particular in ageing animals. Nevertheless, auditory thresholds were already raised across all frequencies tested in young *Pls1 KO* animals, and these could not be attributed to significant hair cell losses. Furthermore, the morphological defects of stereocilia in *Pls1* KO are consistent with an abnormal maintenance of their actin content. The reduction in width was variable between stereocilia in an individual bundle and was often more pronounced at the distal end of an individual stereocilium, where barbed ends of actin filaments are located. As there was no drastic difference in the packing of actin filaments within *Pls1* KO stereocilia, their decrease in width must result from a diminution in the number of actin filaments comprising the central core. The defect was not visible at the level of OHC stereocilia, but because their diameter is smaller than those of IHCs, a decrease in their width would have been more difficult to record. There were, however, signs of degeneration of OHC stereocilia in old animals, indicating that plastin 1 is required for the maintenance of stereocilia in both types of hair cells. Interestingly, the het *jerker* mice with reduced levels of espin also show a diminution in width and a distal tapering of IHC stereocilia, but it does not appear to cause any significant hearing loss; in homozygous *jerker* mutants, however, the stereocilia of both IHCs and OHCs fail to widen and elongate and rapidly disappear within the first 2 weeks of life (12). This indicates that although plastin 1 and espin both contribute to the lateral apposition of actin filaments to the central core of stereocilia, they play non-redundant and specific roles in different types of hair cells, and at different stages of their maturation. While espin, espin-like and other proteins such as GRXCR1 (37,38) are essential for the initial increase in width of immature stereocilia, presumably by promoting the lateral apposition of actin filaments within their core, plastin 1 is largely dispensable at this stage; however, plastin 1 has a unique function in maintaining the correct number and perhaps an optimal crosslinking between actin filaments within the stereocilia of mature hair cells.

What is the reason for the loss of hearing sensitivity in *Pls1* KO mice? The noticeably bent stereocilia of IHCs in adult *Pls1* KO mice are suggestive of a loss of rigidity of the stereocilia core, which might alter the efficiency of mechanotransduction. Our measurements of stereocilia stiffness did not reveal any abnormality, but owing to technical limitations, these were only performed on immature hair cells, which do not show yet any obvious structural anomaly. The additional factors that complicate the determination of stereocilia core stiffness are the presence of numerous extracellular links connecting adjacent stereocilia at immature stages (39–41), and the fact that the flexibility of stereocilia at the taper is the main parameter influencing the stiffness of the hair bundle (42). The only noticeable functional defect in pre-hearing OHCs of *Pls1* KO was a small but significant slowing of the fast component of adaptation but an overall greater extent of adaptation of the MET current in response to mechanical deflections of their bundle. While these small changes in adaptation properties are unlikely to be the cause of the hearing loss in *Pls1* KO, it may be an indication that even at a young stage and in the absence of any visible morphological defect, their stereocilia have abnormal structural and mechanical properties. Additionally, the absence of plastin 1 could affect the transport or localization of components of the mechanotransduction machinery. In the brush border microvilli, the absence of plastin 1, espin and villin results in an inability to retain specific proteins in the apical membrane of the cell and a mislocalization of myosin-1a, a molecular motor involved in anchoring microvillar membrane proteins (43). Comparable defects may occur in stereocilia.

The progressive nature of the morphological defects in *Pls1* KO points at a specific function for plastin 1 in the maintenance of the actin content of mature stereocilia. It has been proposed that a rapid distal to basal actin treadmilling that may be dependent on mechanotransduction occurs in the stereocilia of hair cells (44–47) although recent studies suggest that under normal conditions, protein and actin turnover is fairly slow in mature stereocilia, except at their distal tip (48,49) where the barbed ends of actin filaments are located. Nevertheless, there is growing evidence that the actin core of stereocilia is dynamically remodelled and repaired after mechanical damage, and its long-term maintenance requires continuous expression of β-actin and γ-actin (50,51) and fascin-2 (49). Plastin 1 could help maintaining this actin core, by either promoting the addition of new β- and γ-actin subunits to existing actin filaments or by limiting their depolymerization. Among the proteins that could be functionally connected to plastin 1 is Eps8L2, present at the tip of stereocilia of intermediate size in auditory hair cells. The *Eps8L2* KO mice exhibit a progressive thinning and distal tapering of their stereocilia resembling those seen in *Pls1* KO mice and have an early onset age-related hearing loss (52). Abnormal thinning of stereocilia can also be observed in IHC of aged CBA/Ca mice (24-month-old) and in vestibular hair cells of older humans (RT and AF, unpublished data), suggesting that abnormal maintenance or repair of the stereocilia actin core is a common occurrence in ageing hair cells.

Several genes encoding actin-associated proteins have been linked to hereditary forms of deafness (53,54). Interestingly, the human *PLS1* gene maps to 3q23, and this chromosomal region has been associated by linkage analysis to progressive forms of hearing impairment in the elderly (55). The DFNA18 locus, a dominant progressive form of hearing loss, lies in the same region (56), but currently available mapping and linkage analysis data would suggest that it does not encompass the *PLS1* gene. Further studies should be conducted to investigate whether mutations or reduced expression of the *PLS1* gene are indeed linked to age-related hearing loss in humans. If structural properties of stereocilia are affected by the absence of plastin 1, they may also be more sensitive to noise-induced damage. In fact, actin filaments within the core of stereocilia and their rootlets are damaged by over-stimulation (57,58), and the lack of plastin 1 and other actin-associated proteins could exacerbate this process or limit the capacity of stereocilia to repair following intense noise stimulation. With the prospect of easier access to the personal genome, identifying the genes critical for stereocilia maintenance or repair could help ensure that individuals suffering from ‘stereociliopathies’ are better protected from noise exposure. Some types of hearing impairment resulting from abnormal maintenance of the actin cytoskeleton of stereocilia could also be prevented using targeted gene therapies.

## MATERIAL AND METHODS

### Ethics statement

All animal work was performed under the regulated procedures of the British Home Office and with approval of the UCL Animal Ethics Committee and the University of Sheffield Ethical Review Committee.

### Animals

*Plastin1* (*Pls1*) knock-out mice on a C57BL/6 background (International Strain Designation B6.129-Pls1^tm1^/Cnbc) were obtained from a colony generated as described previously (24). The *Pls1* KO was generated by replacing exon 2 of the *Pls1* gene, containing the start codon, by a neomycin resistance gene cassette. Previously published analysis of plastin protein expression in homozygous animals using a pan-fimbrin antibody confirmed the absence of any residual full-length or truncated plastin 1 protein (24). Immunostaining using an affinity-purified polyclonal rabbit anti-plastin 1 antibody (24) in inner ear tissue in the present study also showed a complete lack of plastin 1 in hair cells of homozygous animals (Supplementary Material, Fig. S1).

### Auditory brainstem response recording

Auditory brainstem responses were obtained from *Pls1* KO, heterozygous (het) and wild-type (wt) mice in three age groups: young adult (5–6 weeks old; *n* = 21), 4 months old (*n* = 15) and animals aged 7 to 9 months (*n* = 10). Recordings were made blind to genotype. Animals were anaesthetized and placed in a sound-isolated chamber. Subdermal needle electrodes (Rochester Electro-Medical) were inserted at the vertex (active), mastoid (reference) and with the ground needle electrode in the hind leg. Recordings were obtained using TDT system3 equipment and software (Tucker-Davis Tech., Alachua, FL, USA). Responses to click stimuli and to tone pips at 8, 12, 24, 32 and 40 kHz were recorded and threshold determined by the lowest level at which the ABR waveform could be recognized. Statistical analysis of the results was performed using Tukey–Kramer multiple comparisons test.

### Histology, immunolabelling and electron microscopy

Auditory bullae were isolated and the cochleae exposed. The inner ear tissues were directly perfused with fixative through openings at the apex of the cochlea and via the round and oval windows. The bullae were then immersed in fixative and fixation continued for 2 h or overnight. For fluorescent labelling and immunolabelling, the fixative was 4% paraformaldehyde in phosphate-buffered saline pH 7.4. For scanning and transmission electron microscopy, the fixative was 2.5% glutaraldehyde in 0.1 m cacodylate buffer with 3 mm CaCl_2_, pH 7.3. Following fixation, the bullae were decalcified in 4% EDTA in the respective buffer for 48 h at 4°C.

Immunolabelling was performed on whole mounts or cryosectioned preparations of the cochleae of 6-week (*n* = 20) and 4-month (*n* = 10)-old animals as described in Daudet and Lewis (2005) (59). The primary antibodies used were affinity-purified rabbit anti-plastin 1 (24), guinea-pig anti-αII-spectrin (60), mouse monoclonal anti-myosin-VIIa (Developmental Studies Hybridoma Bank, clone 138.1), mouse monoclonal anti-calbindin D-28K (clone CB-955, Sigma, UK) and mouse monoclonal anti-parvalbumin (P3088, Sigma, UK). Secondary antibodies were Alexa-conjugated goat antibodies (Invitrogen, UK), and F-actin was stained with Alexa-conjugated phalloidin (Invitrogen, UK). Samples were examined using a Zeiss 510 confocal microscope, and images were further processed using ImageJ and Photoshop (Adobe).

Scanning electron microscopy was performed on half-turn segments of the organ of Corti dissected from glutaraldehyde-fixed, decalcified cochleae. The tissue was post-fixed in 1% OsO_4_ for 2 h at room temperature, before processing through the thiocarbohydazide–OsO_4_ repeated procedure (61). Samples were dehydrated, critical point dried, mounted on support stubs with fast-drying silver paint and lightly sputter coated with platinum. They were examined in a JEOL JSM6700F cold field emission microscope operating at 3 or 5 kV.

For TEM, glutaraldehyde-fixed cochleae were immersed in 1% OsO_4_ in 0.1 m cacodylate buffer for 2 h at room temperature. Cochleae were then dehydrated in alcohol and incubated overnight at 4°C in saturated uranyl acetate in 70% ethanol, to stain tissue ‘en bloc’. After completing dehydration, the cochleae were embedded in plastic. Thin sections collected from different turns of the cochlea were stained with uranyl acetate and lead citrate and examined in a JEOL 1200 EXII instrument operating at 80 kV. Digital images were collected with a Gatan camera.

### Image processing and analysis

All digital images were adjusted only for optimal contrast and brightness using Photoshop CS4. Measurements of the depth of the cuticular plate both in OHC and in IHC were made from thin sections. Microscope magnification was calibrated using images of a cross-grating replica. The depth from the apical membrane of the hair cell to the identifiable basal border of the cuticular plate with the underlying cytoplasm was made at three locations in each cell where the entire width of the cuticular plate was exposed. Measurements of the width and height of the stereocilia were made from scanning electron micrographs obtained from cochleae of 13 adult het and 15 *Pls1* KO animals. Samples were tilted in the electron microscope to gain views as close to perpendicular to the stereociliary bundle as possible. The edge-to-edge distance across each longest stereocilium in a bundle at approximately half way along its shank was measured. The length from the point of insertion at the apical membrane to the distal tip was measured (although these measurements are affected by variable foreshortening errors resulting from viewing an elongated object—the stereocilium—from an (unknown) angle; the angle of view was rarely perfectly perpendicular to the stereocilium). All of the stereocilia in 5–8 individual IHCs in the basal coil, the middle of the lower (basal) half of the middle coil (mid-basal) and the middle of the apical half of the middle coil (mid-apical) were measured in micrographs of nominal magnifications of ×5000 and ×10 000. Measurements were made using analySIS (Olympus) software.

Measurements of the distance between actin filaments within IHC stereocilia were performed on one het and two *Pls1* KO animals, using a method similar to that described in Mogensen et al. (2007) (5). Images of longitudinally oriented stereocilia at a magnification of ×25 000–×30 000 were analysed in two regions: the top of the stereocilia (within 300 nm of the tip) and the shaft of the stereocilia. Five stereocilia were selected for each region for both het and *Pls1* KO samples. Fifty measurements of interfilament distance were taken from each stereocilium. Filaments for measurement were selected by placing an arbitrary grid over the images. Ten squares were selected at random, and five measurements of distance between adjacent actin filaments were taken per square. A total of 250 measurements were taken per stereocilia region. Measurement was carried out using the FIJI/ImageJ software (62). T-tests were performed using GraphPad Prism 6 (GraphPad Software, USA).

### Electrophysiological experiments

Inner hair cells (IHCs, *n* = 10) and outer hair cells (OHCs, *n* = 27) from *Pls1* KO and het mice were studied in acutely dissected organs of Corti from postnatal day 5 (P5) to P8 for OHCs and P24 for IHCs, where the day of birth is P0. Cochleae were dissected in normal extracellular solution (in mm): 135 NaCl, 5.8 KCl, 1.3 CaCl_2_, 0.9 MgCl_2_, 0.7 NaH_2_PO_4_, 5.6 D-glucose and 10 HEPES-NaOH. Sodium pyruvate (2 mm), MEM amino acids solution (50×, without L-glutamine) and MEM vitamins solution (100×) were added from concentrates (Fisher Scientific, UK). The pH was adjusted to 7.5 (osmolality ∼308 mmol kg^−1^). All experiments were performed at room temperature (22–24°C) and using 1.3 mm Ca^2+^ in the extracellular solution. Patch clamp experiments were performed using an Optopatch (Cairn Research Ltd, UK) amplifier. Patch pipettes (2–3 MΩ access resistance) were coated with surf wax (Mr. Zogs SexWax, USA) to minimize the fast capacitance transient of the patch pipette. Data acquisition was controlled by pClamp software using a Digidata 1440A interface (Molecular Devices, USA). Recordings were low-pass-filtered at 2.5 kHz (8-pole Bessel), sampled at 5 kHz and stored on computer for off-line analysis (Origin: OriginLab, USA).

Voltage and current recordings from IHCs were performed as previously described (25) using the following intracellular solution in the patch pipette containing (in mm): 131 KCl, 3 MgCl_2_, 1 EGTA-KOH, 5 Na_2_ATP, 5 HEPES-KOH and 10 Na_2_-phosphocreatine (pH 7.3; osmolality ∼296 mmol kg^−1^). Membrane potentials in voltage clamp were corrected for the voltage drop across the uncompensated residual series resistance (*R*_s_: 0.8 ± 0.1 MΩ, *n* = 10, after up to 80% compensation) and for a liquid junction potential (–4 mV).

Mechanoelectrical transducer currents were elicited by stimulating the hair bundles of OHCs using a fluid jet from a pipette (tip diameter 8–10 µm) driven by a piezoelectric disc (26,27). The pipette tip of the fluid jet was positioned near to the bundles to elicit a maximal MET current. Mechanical stimuli were applied as force-steps or saturating 50-Hz sinusoids (filtered at 0.25 kHz, 8-pole Bessel) with driving voltages of ±40 V. MET currents were recorded with a patch pipette solution containing (in mm): 106 Cs-glutamate, 20 CsCl, 3 MgCl_2_, 1 EGTA-CsOH, 5 Na_2_ATP, 0.3 Na_2_GTP, 5 HEPES-CsOH, and 10 sodium phosphocreatine (pH 7.3). Membrane potentials were corrected for the liquid junction potential (–11 mV). Voltage clamp protocols are referred to a holding potential of –81 mV. The peak MET current–voltage curves (Fig. 8C) were fitted according to a simple single-energy-barrier model (28):

(1) $$\;I(V)=k\{\exp[(1-g)(V-V_{r})/V_{s}]-\exp(-g(V-V_{r})/V_{s})\}$$

where *k* is a proportionality constant, *V*_r_ is the reversal potential, *V*_s_ is a measure for the steepness of the rectification and *γ* is the fractional distance of an energy barrier within the membrane's electrical field, as measured from the outside.

Bundle motion during fluid-jet stimulation was determined by projecting an image of the OHC bundle onto a pair of photodiodes (LD 2–5; Centronics, Newbury Park, CA, USA) at ×360 total magnification as previously described (26). Briefly, the differential photocurrent was filtered at 5 kHz and was calibrated by measuring its amplitude when displacing the photodiodes a known amount in the image plane and then using the magnification to determine the equivalent motion in the object plane. Bundle stiffness was estimated as previously described (28).

Statistical comparisons of means were made by Student's two-tailed *t*-test. Mean values are quoted ±SEM, where *P* < 0.05 indicates statistical significance.

## SUPPLEMENTARY MATERIAL

Supplementary Material is available at *HMG* online.

## FUNDING

This work was supported by the Wellcome Trust (091895 to W.M.), the Biotechnology and Biological Sciences Research Council (BB/FF007175/1 to N.D.), Deafness Research UK (547 to N.D.), the Medical Research Council (87863 to F.R., G1000068 to A.F., R.T. and N.D.) and the Rosetrees Trust (A528 to A.F.); S.L.J. is a Royal Society University Research Fellow. Funding to pay the Open Access publication charges for this article was provided by BBSRC.

## Supplementary Material

Supplementary Data
