# Supplementary material for: Absence of plastin 1 causes abnormal maintenance of hair cell stereocilia and a moderate form of hearing loss in mice
Source: Hum Mol Genet. 2014 Aug 14;24(1):37–49. doi: 10.1093/hmg/ddu417 (PMC4262491; doi:10.1093/hmg/ddu417)
Supplement: Supplementary Data [file supp_24_1_37__index.html]

Absence of plastin 1 causes abnormal maintenance of hair cell stereocilia and a moderate form of hearing loss in mice — Absence of plastin 1 causes abnormal maintenance of hair cell stereocilia and a moderate form of hearing loss in mice — Supplementary Data 

# Absence of plastin 1 causes abnormal maintenance of hair cell stereocilia and a moderate form of hearing loss in mice

## Supplementary Data

Supplementary Data

**Files in this Data Supplement:**

- Supplementary Data - Docx file
