# Supplementary material for: Absence of plastin 1 causes abnormal maintenance of hair cell stereocilia and a moderate form of hearing loss in mice
Source: Hum Mol Genet. 2014 Aug 14;24(1):37–49. doi: 10.1093/hmg/ddu417 (PMC4262491; doi:10.1093/hmg/ddu417)

Supplemental Files legends

Supplemental Figure 1: Plastin 1 expression in the organ of Corti of wild-type and *Pls1* KO mice. Note the lack of plastin 1 immunoreactivity in the stereocilia and cuticular plate of hair cells in *Pls1* KO samples, confirming the absence of plastin 1 expression in this knock-out mouse model.

Supplemental Figure 2: SEM micrographs of the stereociliary bundles of IHCs in *Pls1* KO animals of different ages. At P12, some stereocilia look slightly thinner and shorter than their neighbours (arrowheads), but there is relatively little variability in the morphology of stereocilia within the same row. At 1 month (1 mo) and 3 months (3 mo), there is increasing variability in the width and length of stereocilia within the same row. Note that the stereocilia exhibiting a clear reduction in their diameter and a distal tapering can be either shorter (arrowheads) or longer than normal (arrows).


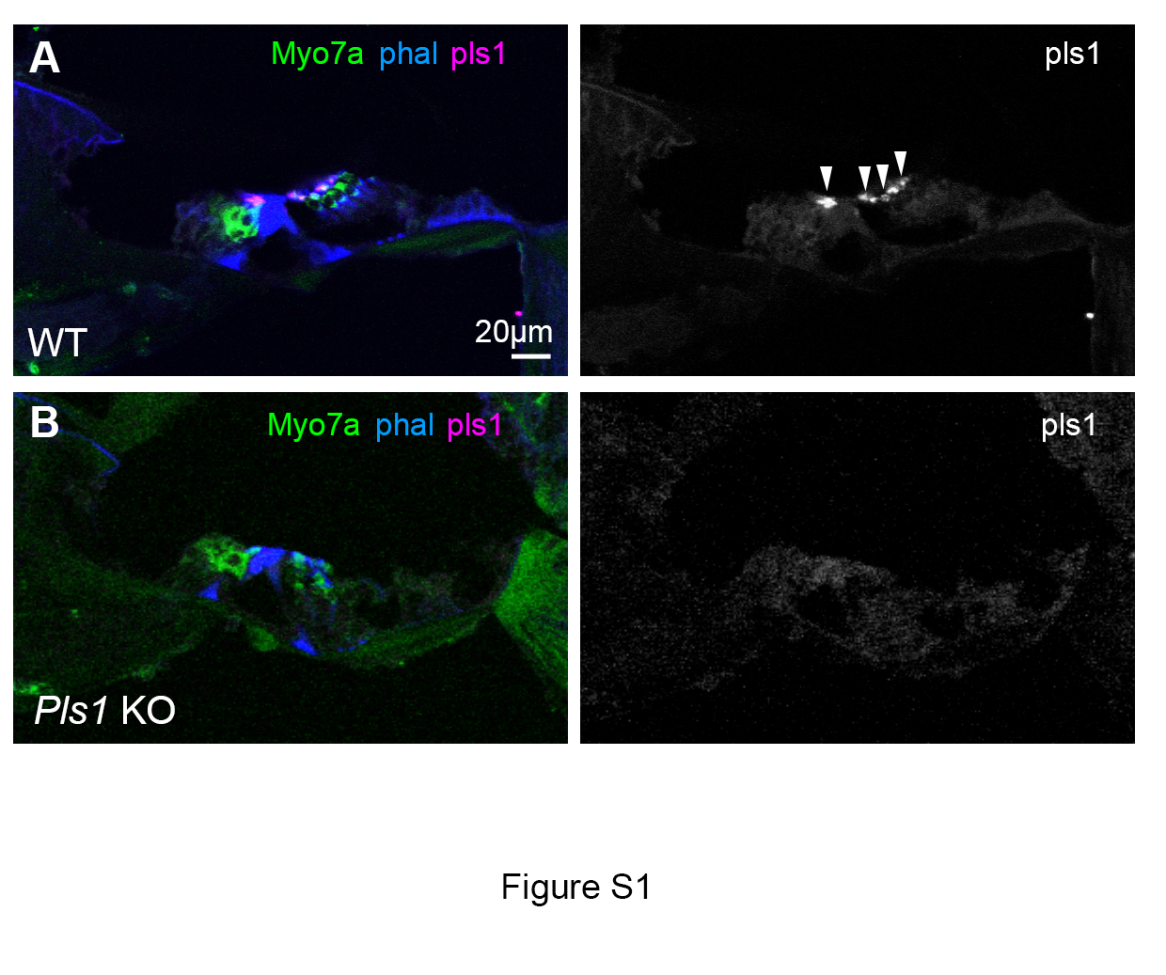


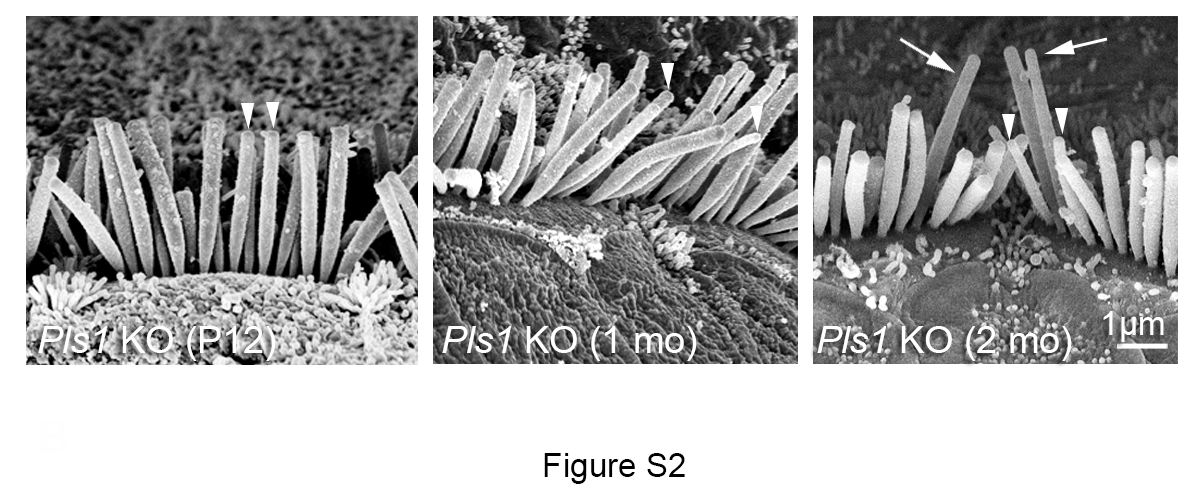

Supplement: Supplementary Data [file supp_ddu417_ddu417supp.docx]
